# Intralesional Bleomycin for Orbital Venolymphatic Malformation

**DOI:** 10.7759/cureus.75613

**Published:** 2024-12-12

**Authors:** Kit May Chow, Kala Sumugam, Amali Ahmad

**Affiliations:** 1 Department of Ophthalmology, Hospital Kuala Lumpur, Kuala Lumpur, MYS; 2 Department of Radiology, Hospital Kuala Lumpur, Kuala Lumpur, MYS

**Keywords:** failed sclerotherapy, intralesional bleomycin, orbital venolymphatic malformation, ovlm, recurrent ovlm

## Abstract

This retrospective case series evaluates the use of intralesional bleomycin injections in treating orbital venolymphatic malformations (OVLM). Three patients, a 7-year-old girl, a 37-year-old woman, and a 56-year-old man, presented with OVLM where the first two were recurrent cases with a history of failed sclerotherapy. All patients received multiple doses of intralesional bleomycin injections, resulting in significant reductions in lesion size, decreased proptosis, and pain relief. Although complete symptom resolution was not achieved, the injections were well-tolerated, and no ophthalmic or systemic side effects were reported. The findings suggest that bleomycin, a sclerosing agent inducing local inflammation and thrombosis, is an effective and safe treatment for OVLM, particularly in cases where excision is not feasible or previous therapies have failed. Intralesional bleomycin injections may offer a valuable alternative for managing this challenging condition.

## Introduction

Orbital venolymphatic malformations (OVLM) are characterized by anomalous vascular and lymphatic structures, often present with proptosis, visual disturbances, and cosmetic concerns. Conventional treatment modalities, such as surgical excision, carry risks of bleeding, nerve damage, and incomplete resection. In a study by Russin et al. including eight patients, the recurrence rate of orbital lymphangiomas after surgery was 71.4% [1]. As such, there has been a growing interest in exploring alternative approaches to manage OVLM, including intralesional bleomycin therapy [2]. This case series aims to highlight that intralesional bleomycin can be effective as a modality of treatment in patients who have already undergone previous sclerotherapy with other substances, and the safety and efficacy of this treatment modality.

This case series was presented as a scientific poster at the Malaysian Oculoplastic Conference 2024 in Penang, Malaysia, on 18-19 May 2024.

## Case presentation

Case 1

**Figure 1:**
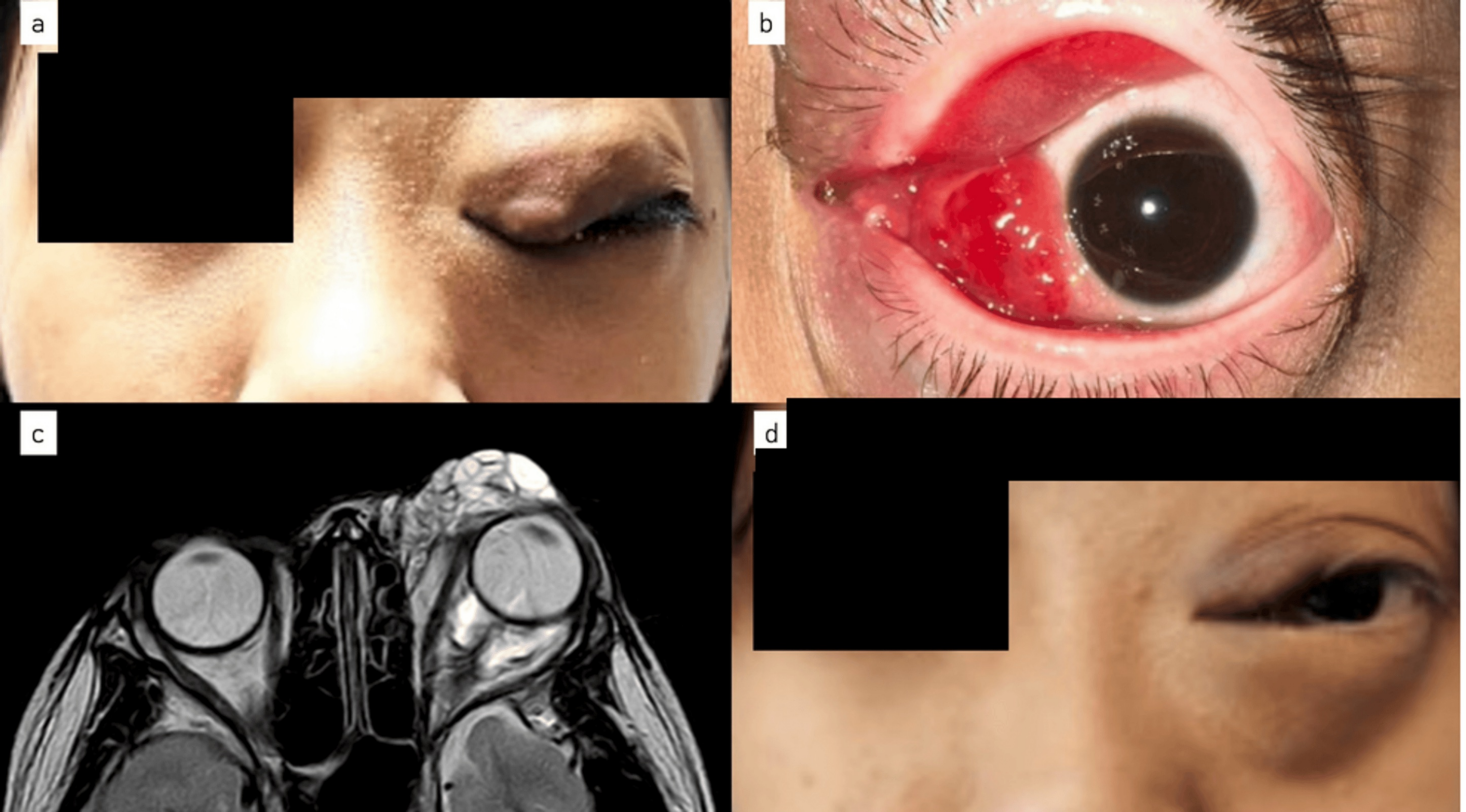
Case 1 on presentation showing left eye proptosis (a) with lesions involving the upper and lower lid (b). MRI orbit showing multiloculated lesions extending into the medial extraconal and retro-orbital space (c). Six weeks post the third intralesional bleomycin showed a good response to treatment with a noticeable decrease in lid swelling (d).

An 11-year-old girl with a history of resolved left OVLM with sclerotherapy done three years ago presented again with increasing left eye proptosis (Figure 1). Magnetic resonance imaging (MRI) orbit showed large multiloculated cystic lesions involving the superior and inferior eyelid, extending into medial extraconal and retro-orbital space with encasement of the intraorbital segment of the left optic nerve. She was given intralesional bleomycin 3IU/mL injected under general anesthesia, repeated three times, six weeks apart.

Case 2

**Figure 2:**
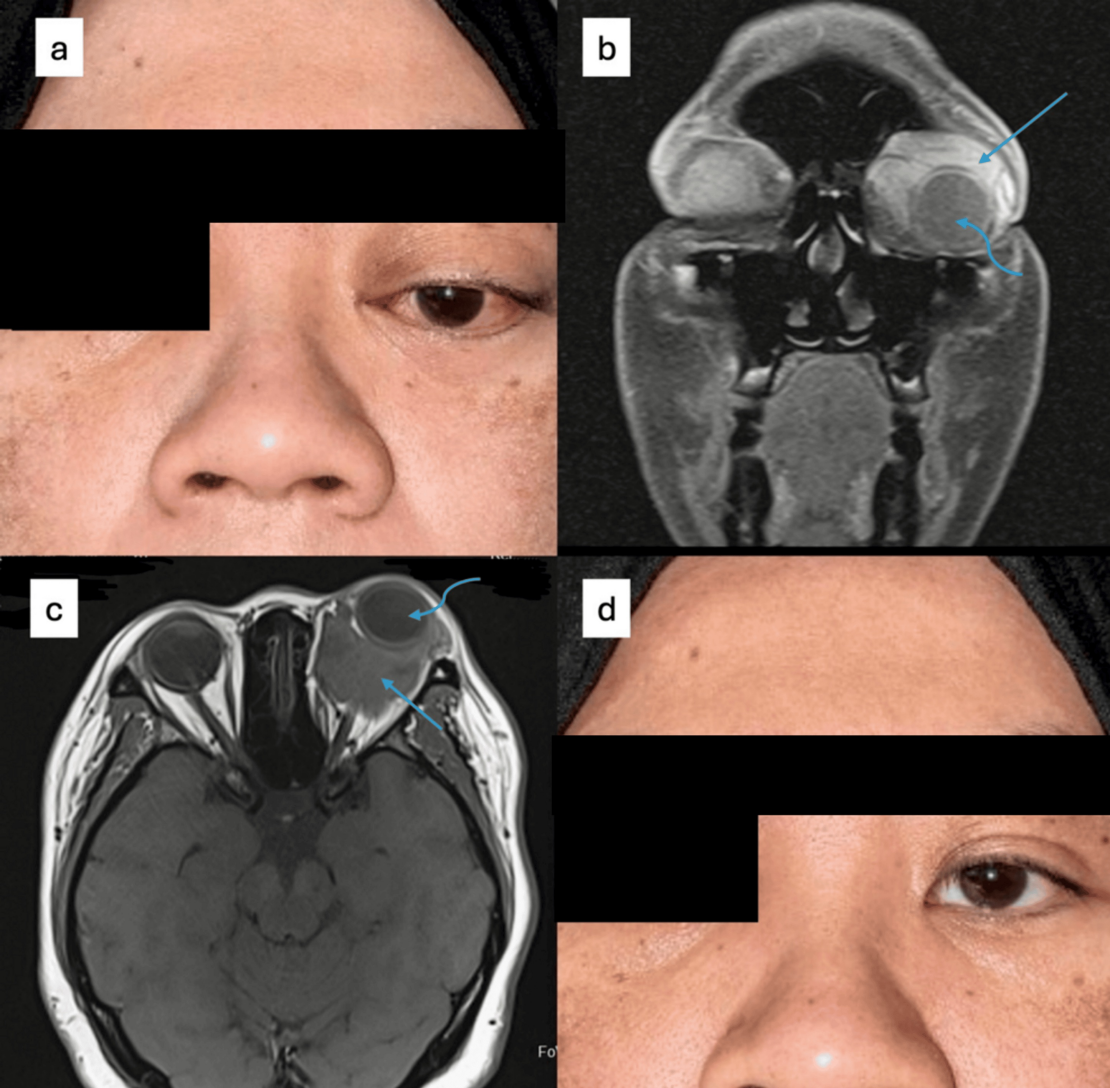
Case 2 on presentation showing proptosis and downward displacement of the left globe (a). Coronal (b) and axial (c) views of MRI T1 images show left OVLM (straight arrow) displacing the left globe (curved arrow) anteroinferiorly). An excellent response was seen one month post the second intralesional bleomycin (d). OVLM: orbital venolymphatic malformation

A 40-year-old lady, with a history of left OVLM with sclerotherapy done twice three years ago, presented with a recurrence of left OVLM with optic nerve compression (Figure 2). She had difficulty sleeping due to pain for which she was referred to the pain management team, but not much relief was attained. She had an excellent response after two doses of intralesional bleomycin, six weeks apart.

Case 3

**Figure 3:**
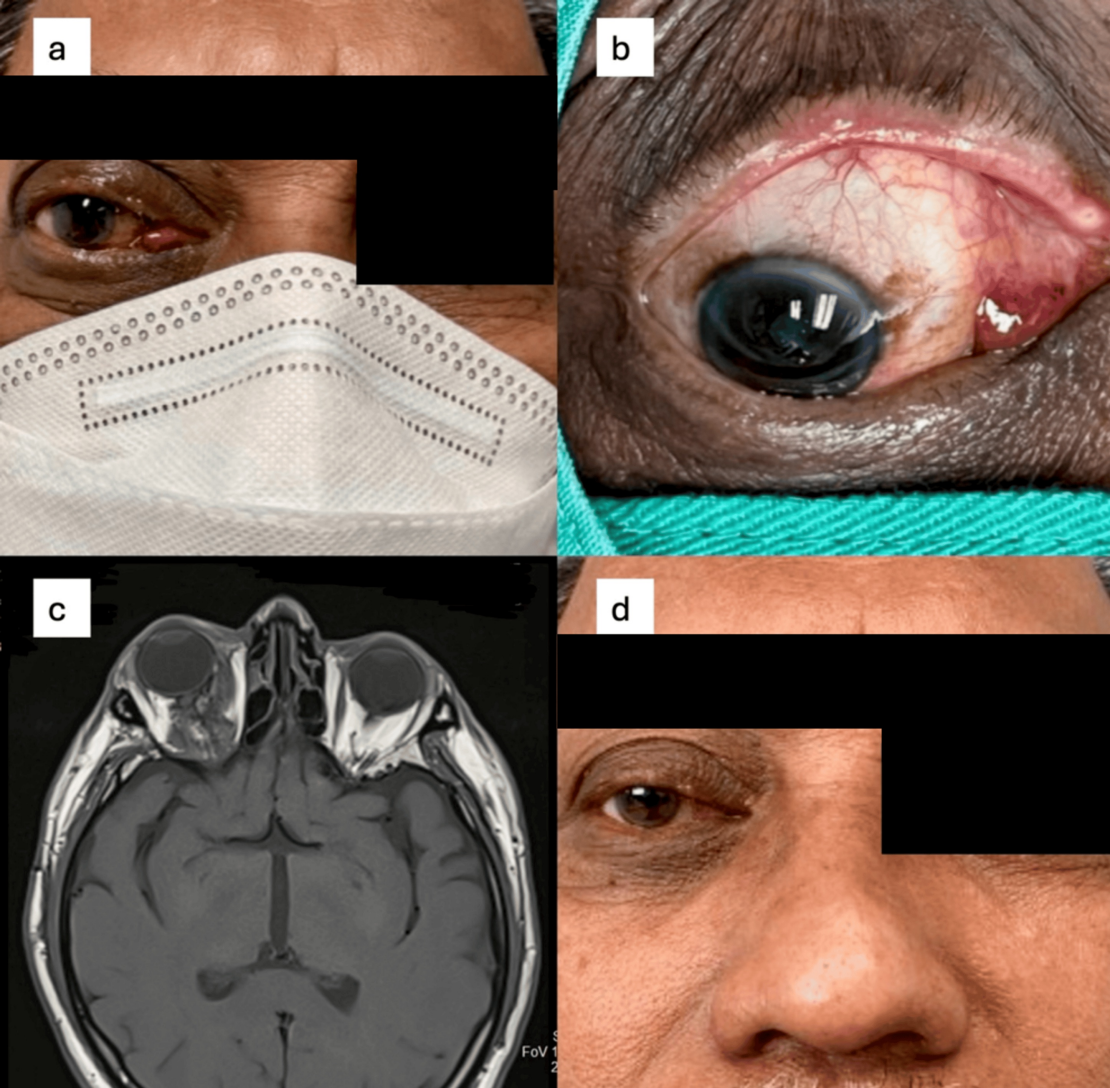
Case 3 on presentation showing right eye exotropia, proptosis, and conjunctiva chemosis medially (a). The eye was also displaced downward and laterally (b). MRI T1 axial showing a heterogenous extraconal/intraconal mass (c). Three months post the second intralesional bleomycin, the patient showed significant regression of the proptosis and conjunctival component (d).

A 56-year-old man presented with right painful proptosis due to an intralesional bleed of the right OVLM (Figure 3). Examination showed right eye exotropia with proptosis. The eye was displaced downward and laterally (Figure 3b). There was a right superomedial mass, and the conjunctiva had chemosis medially. MRI T1 axial showed heterogenous extraconal/intraconal mass in the right orbit (Figure 3c). There was the presence of multiple cystic spaces, indicating a recent bleed, but no extension to the optic canal. The optic nerve was preserved.

Technique

The procedure was performed in the operating room under ultrasound guidance, along with a radiologist. General anesthesia was used for the 11-year-old child while local anesthesia was used for the adults.

**Figure 4:**
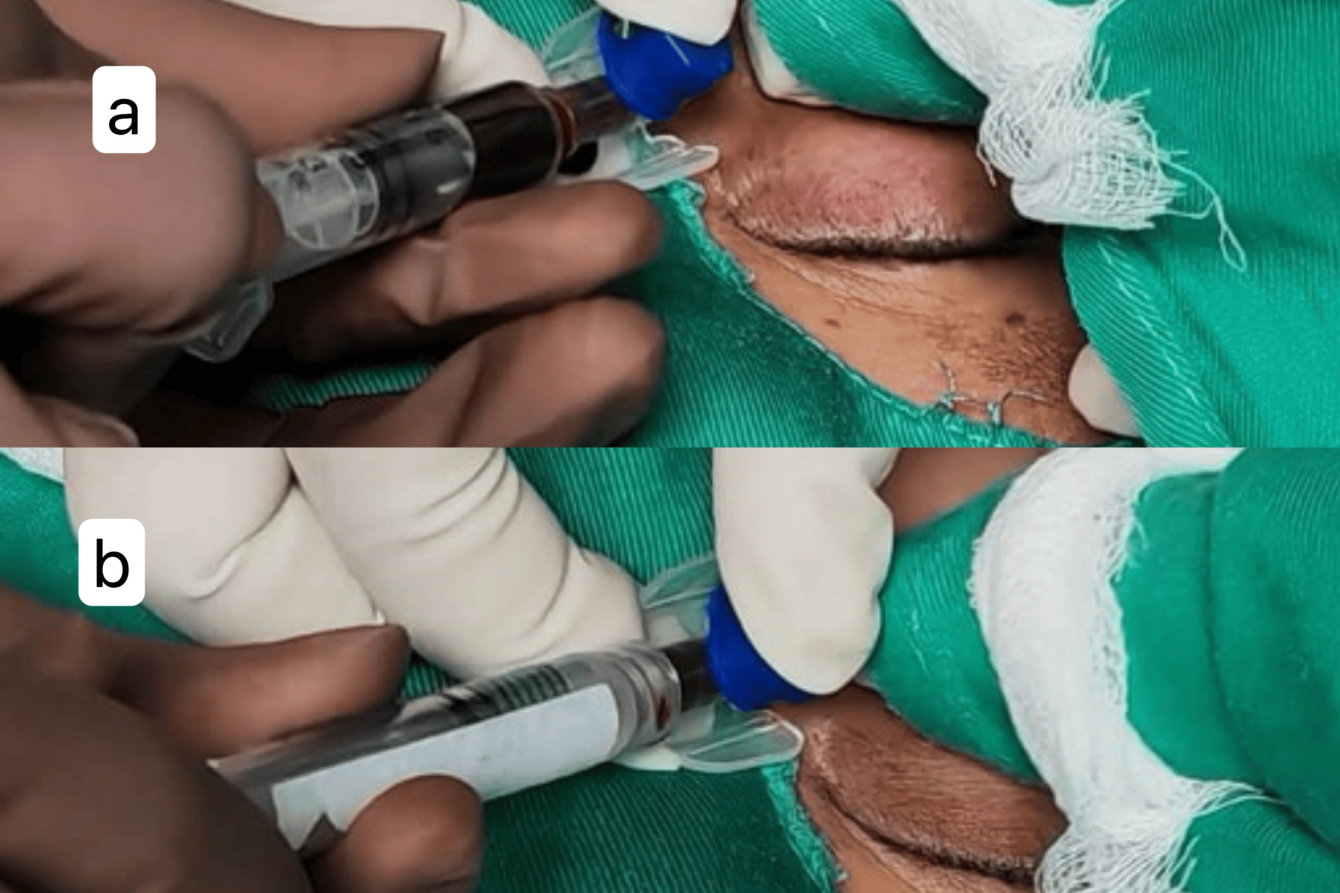
Aspiration of blood from the macrocyst (a) followed by an injection of bleomycin 3 IU/ml (b)

**Figure 5:**
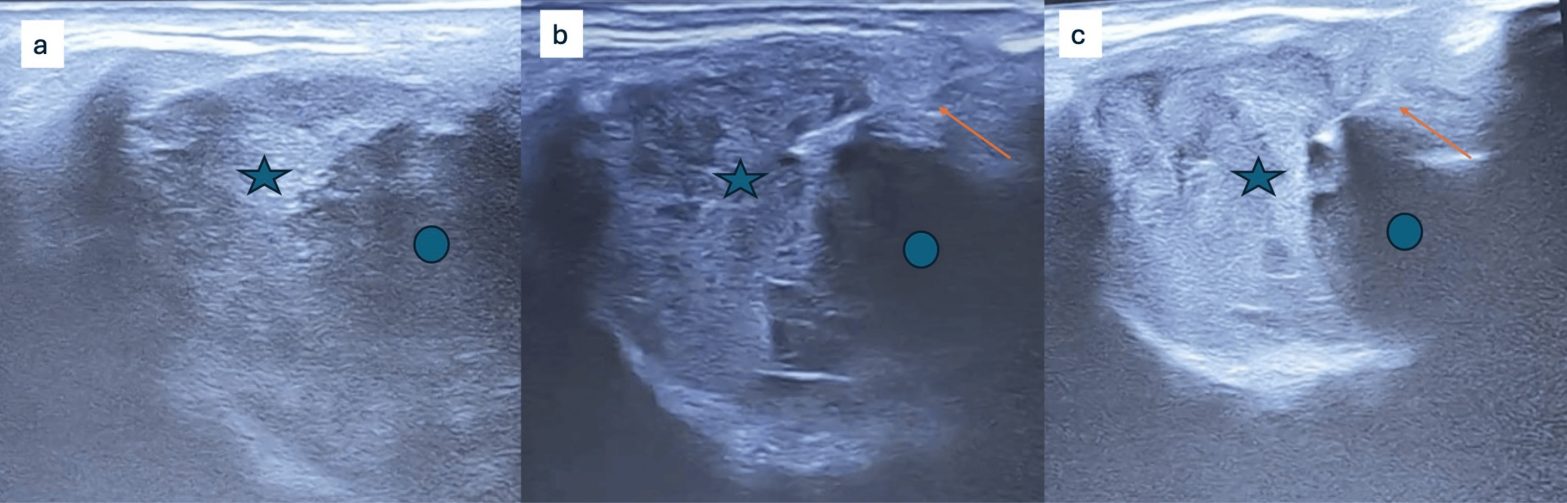
Static ultrasound images before (a, b) and after (c) intralesional bleomycin injection. The lesion (star) appears heterogenously isoechoic before injection (a,b) and hyperechoic after injection (c). It is displacing the globe (round). *The needle is marked as a straight arrow.

As shown in Figure 4, a 23G butterfly cannula was percutaneously inserted into the lesion under ultrasound guidance until blood appeared in the syringe. Blood was aspirated from the microcyst prior to injection to decompress the lesion, reducing the risk of rupture and extravasation. Bleomycin 3 IU/ml was then injected into the lesion. Figure 5 shows static ultrasound images before and after intralesional bleomycin injection. After the injection was completed, a compression bandage was applied, and the patient was observed post-procedure for local or systemic complications.

## Discussion

Bleomycin belongs to the subfamily of glycopeptide antibiotics. It is utilized primarily as a cytostatic antineoplastic agent that acts on the target of the rapamycin (mTOR) pathway. It stops or slows the growth of cancer cells and other rapidly growing cells by damaging their DNA. It exerts a therapeutic effect by inducing endothelial damage within the aberrant vascular channels of the malformation, leading to thrombosis and subsequent fibrosis [3].

Bleomycin sulfate is a lipophilic powder equivalent to 15000 IU. Each vial contains 55-70% bleomycin A and 25-32% bleomycin B. Intralesional bleomycin therapy has emerged as a promising non-surgical option for the management of OVLM. All three patients in this case series responded well to treatment and did not experience any side effects.

Studies have demonstrated favorable outcomes with intralesional bleomycin, including reduction in lesion size, improvement in proptosis, and resolution of symptoms [4]. All the patients in this report experienced all three effects. Its minimally invasive nature makes it an attractive option, particularly for lesions located in surgically challenging areas or for patients with high surgical risks [5].

Despite its potential benefits, intralesional bleomycin therapy is not without limitations. Adverse effects, such as local inflammation, pain, and tissue necrosis, have been reported [6], albeit infrequently. A short course of oral steroids of inflammatory dose post-procedure can minimize the local inflammation. All three patients tolerated the injections with minimal side effects. The optimal dosing regimen and long-term efficacy of bleomycin for OVLM remain areas of ongoing investigation. The cumulative lifetime dose of bleomycin should not exceed 400 mg or 5 mg/kg to limit the risk of pulmonary fibrosis.

Additionally, careful patient selection and thorough evaluation are imperative to ensure safety and efficacy, as certain factors, such as lesion size, location, and proximity to critical structures, may influence treatment outcomes.

## Conclusions

Intralesional bleomycin therapy represents a promising therapeutic option for the management of orbital venolymphatic malformations. While surgical approaches remain a cornerstone of treatment, the minimally invasive nature and favorable outcomes associated with bleomycin make it a valuable adjunct or alternative, particularly in select cases where surgery may be contraindicated or associated with high risks.

Continued research efforts aimed at optimizing dosing regimens, elucidating long-term outcomes, and refining patient selection criteria are warranted to further define the role of intralesional bleomycin in the management of OVLM.
